# Multidimensional poverty in Scotland and health across adulthood—the paradoxical associations with food, fuel, and financial insecurity in later life

**DOI:** 10.1093/eurpub/ckag089

**Published:** 2026-06-19

**Authors:** Sarah N Champagne, Euan Phimister, Aravinda Meera Guntupalli

**Affiliations:** Biostatistics and Health Data Science, Institute of Applied Health Sciences, School of Medicine, Medical Sciences and Nutrition, University of Aberdeen, Aberdeen, United Kingdom; Department of Economics, Business School, University of Aberdeen, Aberdeen, United Kingdom; African Centre for Development Finance, Stellenbosch Business School, Stellenbosch University, Stellenbosch, South Africa; Biostatistics and Health Data Science, Institute of Applied Health Sciences, School of Medicine, Medical Sciences and Nutrition, University of Aberdeen, Aberdeen, United Kingdom

## Abstract

Little is known about the impact that older age and the cost-of-living crisis have on health and wellbeing in Scotland, a country with the lowest life expectancy in Western Europe. Using Scotland as a case study, we aim to capture multidimensional poverty across adulthood and study the associations with health. Relying on the Joseph Rowntree Foundation’s 2023 Poverty in Scotland Survey, we measured the odds of self-reported food, fuel, and financial insecurities across adulthood. We then assessed the association between these insecurities and self-reported physical, mental, and social domains of health. Overall, older adults (aged 65+) report less food, fuel, and financial insecurity. However, lower-income adults aged 35–74 reported significantly higher odds of fuel insecurity compared to younger, upper-income adults. All dimensions of insecurity are significantly associated with higher odds of poor health outcomes, especially among younger adults. Older adults paradoxically report largely fewer negative health impacts despite existing evidence of the group’s health vulnerability to these insecurities. This study shows the importance of analysing poverty multi-dimensionally. It evidences that while pensions are associated with lower reported levels of food and financial insecurity, these associations do not maintain for fuel insecurity, especially among lower-income groups. The results support the reintroduction of non-means-tested winter heating benefits for older adults in Scotland. All three insecurities impact self-reported health, yet reports from older adults do not mirror health across the life course. This may be influenced by survey administration, question framing, understandings of health, problematization of insecurities and stigma.

## Introduction

Scotland has the lowest life expectancy in the United Kingdom (UK) and the highest life expectancy inequality in Western Europe [1, 2]. The combined effects of the COVID-19 pandemic and the ongoing UK cost-of-living crisis have introduced additional economic and social pressures that disproportionately affect people with lower socioeconomic status, potentially worsening life expectancy outcomes in Scotland [3]. Despite growing concern about widening inequalities, relatively little is known about how age structures socioeconomic vulnerability and health in this context.

In response to persistently high poverty rates, the Scottish Government has adopted a multidimensional approach to addressing poverty [4]. Although income remains a central marker of deprivation, focusing solely on income can obscure variations in material conditions, health, and social inclusion, whereas multidimensional measures of poverty can capture heterogeneous and cumulative disadvantages that shape lived experience [4]. Beyond financial insecurity–defined as insufficient monetary means to afford necessities—Scotland has been increasingly focusing on food insecurity, the inability to access sufficient, culturally appropriate food in socially acceptable ways, and fuel insecurity, the inability to power household appliances and adequately heat and/or cool one’s home to maintain physical comfort [5–7]. Examining these insecurities across adulthood may provide deeper insights into the drivers of Scotland’s widening socioeconomic mortality gap.

Older adults face distinct challenges related to both food and fuel insecurity. Barriers to accessing healthy, affordable food, such as mobility constraints and physiological vulnerability, are well-documented [8–11]. In parallel, older adults often have heightened fuel needs due to higher odds of chronic conditions and comorbidities, reduced subcutaneous fat, potentially more time spent at home, and reduced movement [12–14]. Between 2020 and 2023, 15% of pensioners in Scotland experienced relative poverty after housing costs [15]. However, the prevalence of food and fuel insecurity among this population remains less well-documented.

While financial insecurity has been extensively studied and associated with adverse health outcomes such as shorter life expectancy, other dimensions of poverty may exert distinct or compounding effects on dimensions of health and wellbeing in later life [12, 13, 16, 17]. These effects may be shaped by cumulative disadvantage across the life course, creating conditions in which insecurities both worsen health and are exacerbated by pre-existing health problems [18]. It is essential to unpack heterogeneous experiences across adulthood to highlight vulnerable groups and mitigate this threat. Using Scotland as a case study, we examine multidimensional poverty among adults, measured through self-reported food, fuel, and financial insecurity during the cost-of-living crisis, and investigate how these insecurities are associated with health outcomes.

## Methods

### Data source and sample

We relied on data from the latest phase of the Joseph Rowntree Foundation’s (JRF) “Poverty in Scotland” survey, conducted online with community-dwelling adults aged 18–75+ between 19 and 29 March 2023.

The survey yielded 4203 participants. Data were anonymized, weighted, and designed to be representative of Scotland by age, sex, ethnicity, region, and social grade. Missing responses were excluded from the analysis; missing values ranged from 0.3% for ethnicity to 1.0% for financial insecurity.

### Survey methods and questions

In this study, we operationalize multidimensional poverty using three indicators, food, fuel, and financial insecurity, selected to capture distinct but interrelated domains of material hardship that may be particularly salient in later life. Rather than combining these into a composite multidimensional poverty index, we model each dimension separately to preserve information on their potentially heterogeneous associations with health outcomes across adulthood. Constructing an index (e.g. following Alkire-Foster or related frameworks) would require normative weighting and aggregation assumptions that are unlikely to be stable across the life course and cannot be robustly specified given the available data [19].

This study draws on self-reported food, fuel, and financial insecurities, which, unlike expenditure-based indicators, capture lived experiences such as self-rationing, reliance on community support, and other non-monetary coping strategies, providing a more nuanced picture of insecurity [20]. Similarly, the study uses self-reported health measures, which are recognized as reliable indicators in population health research [21].

Health outcomes captured the self-reported physical, mental, and social health impacts of the cost-of-living crisis. Each outcome was recorded as binary indicators capturing negative impacts.

Food and fuel insecurity were derived from self-reported behavioural coping strategies, consistent with experience-based approaches that capture constraints in meeting basic needs and, despite potentially reflecting both temporary and more sustained hardship, are widely used to represent the lived experience of insecurity [22, 23]. The measures were binary (yes/no) and referenced behaviours adopted in response to the cost-of-living crisis. Participants were asked: “Which, if any, of the following have you done to reduce spending on household expenses as a result of the higher cost of living?”

Food insecurity was coded as present if respondents reported (1) skipping meals, (2) reducing meal size, or (3) accessing a food bank. Fuel insecurity was coded as present if respondents (1) heated their home less or less often than they needed to, or (2) were behind on electricity or gas bills. Financial (in)security was assessed using a 0–10 self-reported financial security scale, where 0 indicated severe insecurity (i.e. struggling to afford essentials on a day-to-day basis) and 10 indicated very secure (i.e. saving, investing or spending on non-essentials) in the past 12 months. A score of 0–3 was categorized as “financially insecure.”

### Control variables

Income was measured as monthly equivalized before housing costs (BHC) and categorized into low (<£2063), middle (£2063–£4000), and high (>£4000) income groups. Because only 8.2% of participants were in the high-income category, middle- and high-income respondents were combined into an “upper-income” category. Although relative poverty after housing costs is the primary indicator used in Scotland, BHC measures follow similar trends across the adult age range [15].

The JRF survey asked respondents if they identify as male, female, in another way, or prefer not to say. Given the choice of terms, the survey measured sex (biological and bodily processes and characteristics) and rather than gender identity (“a person’s innate, deeply felt internal and individual experience of gender”) [24]. Similarly, ethnicity was excluded from regression analyses due to small sample sizes among racially minoritized respondents, particularly in later life, limiting statistical reliability.

The survey assessed presence of physical disabilities, mental health conditions, and long-term physical health conditions affecting respondents or household members. These variables were included as controls in the multidimensional health models because our aim was not to measure current health status, but rather to isolate the association between age, multidimensional insecurity, and reported health impacts. We also conducted sensitivity analyses excluding these controls (Supplementary Appendix 2).

### Weights

Analytical weights were applied so that the sample matched Scottish population distributions. Weights were provided by Savanta ComRes, a market research agency, to the JRF based on age, sex, region, ethnicity, and social grade.

### Statistical analysis

We first estimated the prevalence of food, fuel, and financial insecurity across age groups. We then conducted two sets of multivariable logistic regression analyses:

- three models predicting food, fuel and financial insecurities, including an interaction between age group and income category; and
- three models predicting negative physical, mental and social health impacts, with age and security status in interaction with food, fuel, and financial insecurities measures.

In all models, secure adults aged 18–24 served as the reference group. Models adjusted for sex, solo-dweller status, and location. The health models also adjusted for pre-existing health conditions.

## Results

Nearly one in four respondents (23.4%) were older adults, slightly above the Scottish Census 2022 estimates, (20.1% aged 65+). The sample was 51.5% female, closely matching census proportions (51.4%). Respondents represented all regions of Scotland, with Glasgow and Edinburgh separated from the broader region. The majority identified as white (95.4%), slightly higher than census estimates (92.9%). These slight variations were likely due to the weighting being conducted before the 2022 census results were published. For more information on sociodemographic characteristics, see Supplementary Appendix 1.

**Figure 1.**
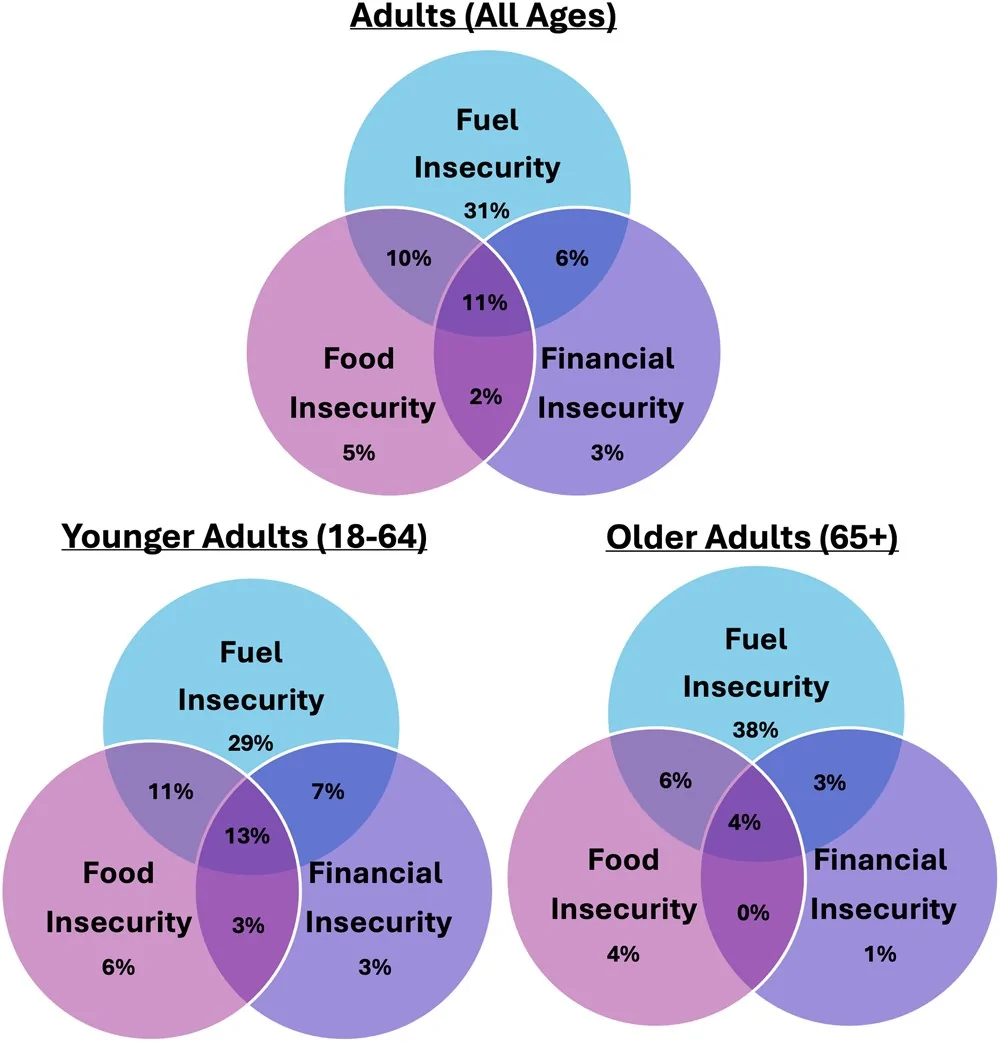
Prevalence and congruence of food, fuel, and financial insecurity among all, younger, and older adults. Three Venn diagrams show the prevalence and overlap of food, fuel, and financial insecurity among all adults, younger adults aged 18–64, and older adults aged 65 and over.

Figure 1 depicts that fuel insecurity was the most common form of deprivation among survey participants. Across all adults, 58% reported fuel insecurity, 28% food insecurity, and 22% financial insecurity following the cost-of-living crisis. All three dimensions of poverty were reportedly experienced at higher rates among adults 18–64 than among older adults. Eleven percent of respondents reported experiencing all three insecurities, underscoring the multidimensional nature of poverty.

**Figure 2.**
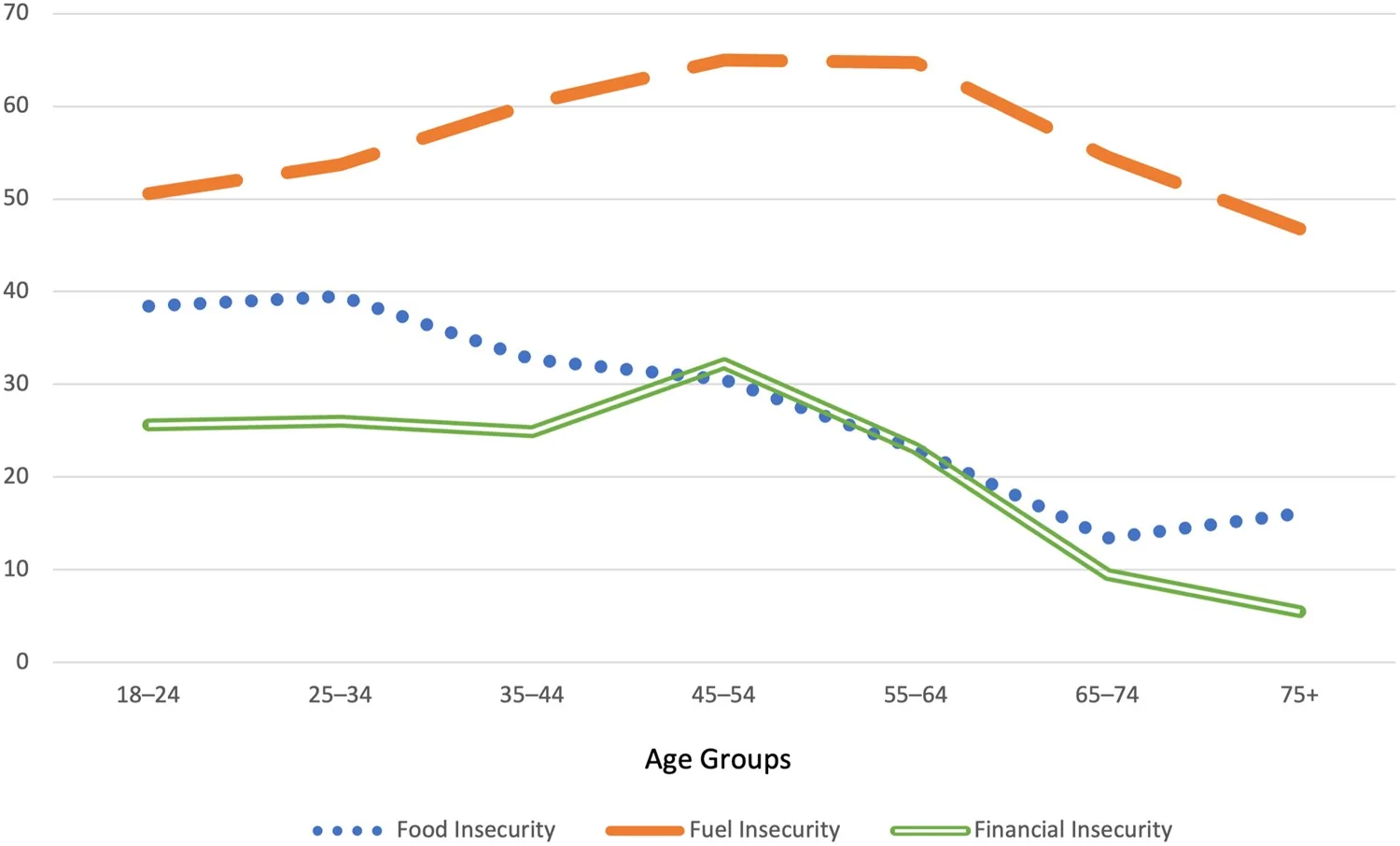
Prevalence of food, fuel, and financial insecurity across age groups. Line chart showing food, fuel, and financial insecurity by age group.

Fuel insecurity displayed a non-linear relation with age, as shown in Fig. 2. Nearly 50% of adults aged 18–24 reported fuel insecurity, rising to 65% among adults aged 45–64, before declining among those aged 65+. Financial insecurity showed a similar non-linear pattern, peaking at ages 45–54, whereas food insecurity declined steadily with age, aside from a small increase among adults 25–34 and 75+.

Table 1 presents the logistic regression results estimating the odds of experiencing food, fuel, and financial insecurity across age and income groups in response to the cost-of-living crisis. Lower-income adults aged 35–74 had significantly higher odds of fuel insecurity compared with upper-income adults aged 18–24 [e.g. odds ratios (ORs) ≥1.64]. By contrast, upper-income adults aged 55+ had significantly lower odds of food insecurity (ORs ≤ 0.47) while lower-income adults aged 18–54 had substantially higher odds (ORs ≥2.68). For financial insecurity, a clear gradient was evident: upper-income older adults (particularly those aged 65+) reported significantly lower odds, whereas lower-income adults aged 18–64 had markedly greater odds of experiencing financial insecurity (ORs ≥3.38).

**Table 1.** Logistic regression with dimension of poverty as the outcome variable and age and lower-income in interaction

	Fuel insecurity		Food insecurity			Financial insecurity	
	Odds ratio	[95% conf. interval]	Odds ratio		[95% conf. interval]	Odds ratio	[95% conf. interval]
Income status by age							
Upper-income 18–24	1.00		1.00			1.00	
Upper-income 25–34	1.01	[0.67–1.52]	1.24	[0.79–1.93]		1.25	[0.70–2.21]
Upper-income 35–44	1.34	[0.88–2.04]	0.87	[0.55–1.39]		1.08	[0.60–1.94]
Upper-income 45–54	1.31	[0.87–1.97]	0.72	[0.46–1.13]		1.49	[0.86–2.60]
Upper-income 55–64	1.33	[0.88–2.00]	0.47^a^	[0.29–0.77]		0.93	[0.52–1.67]
Upper-income 65–74	0.92	[0.61–1.39]	0.19^a^	[0.11–0.32]		0.24^a^	[0.12–0.48]
Upper-income 75+	0.77	[0.50–1.21]	0.41^a^	[0.24–0.70]		0.11^a^	[0.04–0.33]
Lower-income 18–24	0.83	[0.52–1.31]	2.94^a^	[1.81–4.79]		3.38^a^	[1.88–6.08]
Lower-income 25–34	0.95	[0.60–1.49]	3.51^a^	[2.17–5.67]		3.59^a^	[2.01–6.43]
Lower-income 35–44	1.64^a^	[1.02–2.63]	2.72^a^	[1.68–4.43]		4.02^a^	[2.24–7.22]
Lower-income 45–54	2.93^a^	[1.79–4.79]	2.68^a^	[1.67–4.32]		6.89^a^	[3.87–12.25]
Lower-income 55–64	2.60^a^	[1.61–4.20]	1.45	[0.89–2.35]		3.65^a^	[2.05–6.51]
Lower-income 65–74	1.82^a^	[1.05–3.14]	1.24	[0.70–2.20]		1.67	[0.86–3.26]
Lower-income 75+	1.09	[0.57–2.06]	0.95	[0.47–1.91]		1.22	[0.54–2.76]

a Statistically significant, *P* < .05.

Ref, reference group.

Also controlled for sex, solo dweller status, and location.

Fuel, food, and financially secure individuals aged ≥45 years generally reported substantially lower odds of adverse physical and mental health outcomes, with the lowest odds observed among those aged 65+ (e.g. ORs often ≤ 0.30) relative to secure adults 18–24 (Table 2). In contrast, insecurity was consistently associated with elevated odds of negative outcomes, particularly for mental and social health, and most markedly among working-age groups. Food and financial insecurity showed the largest effect sizes on health and wellbeing. Fuel insecurity showed a more moderate pattern, with increased odds of mental and social health impacts concentrated in younger and middle-aged adults. Fuel insecurity associations with negative physical and mental health attenuated in older insecure groups (ORs ≤0.37). Overall, adults earlier in the life course are more likely to report health associations with these dimensions of poverty.

**Table 2.** Logistic regression with health impacts as the outcome and age and insecurity in interaction.

	Negative physical health impact		Negative mental health impact		Negative social impact	
	Odds ratio	(95% conf. interval)	Odds ratio	(95% conf. interval)	Odds ratio	(95% conf. interval)
Fuel security status by age						
Secure 18–24 (ref)	1.00		1.00		1.00	
Secure 25–34	0.65	0.42–1.02	0.74	0.46–1.19	0.99	0.65–1.50
Secure 35–44	0.67	0.41–1.09	0.73	0.44–1.20	1.03	0.66–1.59
Secure 45–54	0.51^a^	0.32–0.79	0.47^a^	0.29–0.75	0.79	0.51–1.20
Secure 55–64	0.30^a^	0.18–0.49	0.22^a^	0.14–0.36	0.47^a^	0.30–0.74
Secure 65–74	0.11^a^	0.06–0.20	0.11^a^	0.07–0.20	0.19^a^	0.11–0.32
Secure 75+	0.17^a^	0.09–0.32	0.14^a^	0.08–0.26	0.24^a^	0.14–0.41
Insecure 18–24	0.91	0.56–1.46	2.49^a^	1.40–4.43	2.14^a^	1.34–3.44
Insecure 25–34	1.30	0.86–1.96	1.96^a^	1.21–3.16	2.54^a^	1.67–3.87
Insecure 35–44	1.19	0.80–1.78	1.65^a^	1.06–2.58	1.90^a^	1.28–2.81
Insecure 45–54	1.08	0.73–1.59	1.21	0.79–1.87	2.12^a^	1.45–3.12
Insecure 55–64	0.85	0.57–1.26	0.82	0.54–1.26	1.46	0.99–2.13
Insecure 65–74	0.32^a^	0.20–0.51	0.35^a^	0.23–0.56	0.73	0.48–1.10
Insecure 75+	0.37^a^	0.22–0.62	0.29^a^	0.17–0.50	0.62	0.38–1.01
Food security status by age						
Secure 18–24 (ref)	1.00		1.00		1.00	
Secure 25–34	1.11	0.74–1.67	0.70	0.46–1.06	1.09	0.75–1.58
Secure 35–44	1.13	0.75–1.71	0.72	0.48–1.07	1.06	0.73–1.52
Secure 45–54	0.93	0.63–1.38	0.48^a^	0.32–0.71	0.92	0.65–1.31
Secure 55–64	0.69	0.46–1.03	0.32^a^	0.22–0.47	0.69^a^	0.48–0.98
Secure 65–74	0.25^a^	0.16–0.40	0.15^a^	0.10–0.23	0.32^a^	0.22–0.48
Secure 75+	0.37^a^	0.22–0.62	0.14^a^	0.09–0.23	0.30^a^	0.19–0.46
Insecure 18–24	3.25^a^	1.96–5.41	1.77	0.96–3.24	2.35^a^	1.43–3.87
Insecure 25–34	2.76^a^	1.76–4.35	1.77^a^	1.08–2.91	2.60^a^	1.68–4.02
Insecure 35–44	3.35^a^	2.09–5.38	2.04^a^	1.20–3.46	2.71^a^	1.72–4.27
Insecure 45–54	3.85^a^	2.44–6.09	2.49^a^	1.48–4.19	5.36^a^	3.27–8.80
Insecure 55–64	4.00^a^	2.38–6.72	1.57	0.92–2.70	3.40^a^	2.04–5.67
Insecure 65–74	1.75	0.90–3.42	0.74	0.39–1.41	1.87^a^	1.03–3.41
Insecure 75+	0.92	0.42–2.02	0.42^a^	0.20–0.89	1.22	0.61–2.41
Financial security status by age						
Secure 18–24 (ref)	1.00		1.00		1.00	
Secure 25–34	0.93	0.64–1.35	0.78	0.53–1.13	1.10	0.79–1.54
Secure 35–44	0.95	0.65–1.38	0.75	0.51–1.09	0.96	0.69–1.34
Secure 45–54	0.71	0.49–1.03	0.49^a^	0.34–0.70	0.82	0.59–1.14
Secure 55–64	0.59^a^	0.41–0.85	0.33^a^	0.23–0.48	0.65^a^	0.47–0.90
Secure 65–74	0.23^a^	0.15–0.35	0.16^a^	0.11–0.23	0.30^a^	0.21–0.43
Secure 75+	0.25^a^	0.16–0.41	0.16^a^	0.10–0.24	0.31^a^	0.21–0.46
Insecure 18–24	2.30^a^	1.35–3.92	3.36^a^	1.52–7.44	2.84^a^	1.58–5.08
Insecure 25–34	2.85^a^	1.76–4.62	2.96^a^	1.58–5.54	3.40^a^	2.03–5.70
Insecure 35–44	2.76^a^	1.72–4.44	3.50^a^	1.87–6.55	4.19^a^	2.47–7.11
Insecure 45–54	2.65^a^	1.72–4.07	2.49^a^	1.49–4.17	5.18^a^	3.20–8.38
Insecure 55–64	2.03^a^	1.22–3.38	1.48	0.88–2.49	2.56^a^	1.57–4.17
Insecure 65–74	0.89	0.44–1.83	1.08	0.55–2.10	2.38^a^	1.17–4.86
Insecure 75+	6.77^a^	1.68–27.27	0.81	0.26–2.56	1.75	0.59–5.18

a Statistically significant, *P* < .05.

Ref, reference group.

Also controlled for sex, solo dweller status, location, and the presence of physical disability, mental health condition, or physical health condition of the respondent or in the household.

In models excluding pre-existing conditions (Supplementary Appendix 2), protective associations of security extended to younger age groups. Additionally, fuel insecure older adults reported decreased odds of negative social impacts (ORs ≤ 0.61) and those in food insecurity reported increased negative physical health impacts (ORs ≥ 2.21).

Finally, although objective measures of food and fuel insecurity were not available, Supplementary Appendix 3 compares self-reported financial insecurity with reported income, illustrating that older adults frequently reported feeling financially secure despite income levels that would typically be classified as financially insecure, potentially explaining several paradoxical observed patterns.

## Discussion

This study provides an up-to-date picture of food, fuel, and financial insecurity among Scottish older adults and the wider adult population following the cost-of-living crisis. Our findings indicate that more than half of Scottish adults reported heating less than they needed to or falling behind on fuel bills, pointing to a high prevalence of unmet energy needs that warrants urgent comprehensive policy action. In contrast, the Scottish Government classifies 34% of households as fuel poor (relying on Office for National Statistics data and measuring fuel poverty as households spending more than 10% of adjusted net income on energy) [25]. While this remains substantial, it raises concerns that the 10% threshold may inadequately capture lived experiences of thermal discomfort, coping strategies, or constrained consumption, thereby underestimating the extent of fuel insecurity [6]. Integrating objective and subjective indicators would provide a more robust assessment of fuel insecurity, unavailable to us with this data set, to further explore the discrepancy. Nevertheless, our results consistently indicated that a large proportion of households experience either unmet heating needs or financial strain related to energy.

The study also reveals a substantial variation in how food, fuel, and financial insecurity overlap. While prior research has shown a close correspondence between financial and food insecurity, our findings indicate that fuel insecurity does not closely align with financial insecurity [26]. Notably, 40% of adults and 44% of older adults reported experiencing fuel insecurity, even in the absence of financial insecurity. Moreover, the non-uniform associations between each insecurity and dimensions of health and wellbeing further underscore the need for a multidimensional approach to assessing health impacts.

### Older age, income status, and prevalence of multidimensional poverty

Food, fuel, and financial insecurities are significantly associated with age, though the associations differ between age groups and income categories. Lower-income middle-aged and older adults (45–74 years) are particularly vulnerable to fuel insecurity. Applying prevalence estimates to national population figures suggests that approximately 520 000 older adults in Scotland may be experiencing fuel insecurity, which is still high despite overall lower insecurity rates in later life [27]. This suggests that Winter Fuel Payments, as granted to pensioners at the time, were likely insufficient to protect lower-income older adults and that the Scottish Government should additionally explore fuel support for lower-income middle-aged populations. This issue is especially salient in light of the UK Government attempts in July 2024 to restrict the Universal Winter Fuel Payment to pensioners receiving means-tested benefits [28]. Following significant political and public pushback, including from the Scottish Government, the policy was subsequently modified, with eligibility re-extended to pensioners earning under £35 000, and the Scottish Government aligning its Pension Age Winter Heating Payment with this revised baseline [28, 29]. These developments underscore the need for more robust and inclusive fuel insecurity support in Scotland, as limiting provision to pensioners alone or older adults on means-tested benefits exclusively would likely have serious public health consequences.

Upper-income older adults seem uniquely protected from financial insecurity. This pattern may reflect the relative stability of upper-income pensions, which, under the UK’s triple lock system (whereby pensions rise in line with inflation, average earnings, or 2.5%, whichever is highest) has offered greater insulation from the cost-of-living crisis than wage-based income [30]. However, higher-income older adults are unlikely to rely on state pensions alone, which may explain why this potential protection does not extend to lower-income older adults [31]. Lower-income adults aged 65–74 remain significantly more likely to experience financial insecurity, mirroring the heightened vulnerability observed among lower-income adults below pension age. These findings suggest that not even the triple lock can protect lower-income older adults from the cost-of-living crisis. They, furthermore, stress the need for targeted support for lower-income adults across the age spectrum.

Our findings reveal a pronounced age–income gradient in food insecurity: lower-income younger adults were substantially more likely to experience food insecurity, whereas upper-income older adults were associated with lower odds of food insecurity. This pattern mirrors evidence of disproportionately higher foodbank use among younger populations [32]. While pensions are not adjusted for inflation specific to food and fuel costs, rates of food insecurity nonetheless track financial insecurity more closely than fuel insecurity [33, 34]. This provides further support for the “cash-first” approach to food security (prioritization of direct emergency cash payments or vouchers over food parcels) currently advanced by the Scottish Government [32]. Although structural factors such as food deserts and limited access to cooking and food storage infrastructure may also contribute to food insecurity, the observed gradient suggests that income adequacy remains a key correlate of food insecurity across the life course [35, 36].

Our findings stress the importance of adopting an intersectional age-income approach to addressing multidimensional poverty across adulthood. Analyses based on age alone obscure the concentration of food, fuel, and financial insecurities among lower-income groups, particularly among middle-aged and older adults. This pattern indicates that vulnerability in later life is not uniform but stratified by income, with substantially different risk profiles across the income distribution. Further research is needed to examine additional dimensions of intersectionality.

### Health associations of multidimensional poverty and the role of age

Older adults who are food, fuel, or financially secure are associated with significantly lower odds of reported negative physical health, mental health, and social wellbeing outcomes compared to secure adults ages 18–24. Among materially insecure adults, younger and middle-aged adults (e.g. 18–54) report higher odds of negative health outcomes. Paradoxically, insecure older adults seem protected from select negative health outcomes. Most notably, fuel insecure older people report lower odds of physical, mental, and social health impacts. This is largely incongruous with the existing evidence, which instead stresses that older people are more vulnerable to the health consequences of these insecurities [11, 14, 17]. That said, no fuel insecure age group reported higher odds of physical health impacts. Self-reported health, while a useful tool to capture overall health, may be less useful for unpacking certain drivers of poor health due to other confounding variables. For example, it may be more difficult to delineate the impact that fuel insecurity is having on one’s physical health when pre-existing conditions and ageing are taking their own tolls, this is particularly salient among the most deprived areas where healthy life is on average 26.2 years lower [37, 38]. Similarly, the questions focus on health impacts attributed to the cost-of-living crisis, which may be difficult for respondents to distinguish from other, overlapping determinants of poor health, such as cumulative socioeconomic disadvantage.

The paradoxical association between materially insecure older adults and negative reported health outcomes may reflect a combination of selection and reporting processes, rather than a true protective effect. Given the low life expectancy (80.8 years for females and 76.8 years for males) and high life expectancy inequality in Scotland (around 59 years for males and females), there is the potential for survivor bias in the health of the sample of older adults [2, 38]. Numerous low-income Scottish adults are more likely to experience health conditions before reaching pensionable age or may not reach it at all, especially among the most deprived communities where life expectancy is 10.5–13.2 years lower [2, 38]. Thus, the older adult sample is likely selectively composed of individuals who are relatively healthier or more resilient than their lower-income peers who experienced earlier morbidity or mortality leading to an underrepresentation of the most disadvantaged life-course trajectories. Many of the 65+ participants may therefore be recently low-income, and as such, our findings may not speak to the compounding impact that multidimensional poverty may have on health with increasing age.

Cohort effects and differing health expectations may further shape how older adults interpret and attribute changes in their health, especially when distinguishing between the effects of insecurity and ageing or pre-existing conditions. Older individuals may recalibrate their expectations around food, fuel, and financial adequacy, leading to a lower likelihood of identifying or reporting insecurity. Qualitative research from England, for example, shows that older adults rarely problematize fuel poverty, instead framing coping practices as “what to do to get by” [39]. Such normalization may be particularly pronounced among cohorts who have experienced prolonged periods without central heating or who have routinely rationed food, potentially contributing to the lower reported prevalence of insecurities among older adults compared to younger groups [40]. Stigma surrounding financial hardship appears to be more pronounced among older generations in the UK, which may further suppress self-reporting of insecurity in survey data such as the JRF study [41, 42]. Together, these mechanisms warrant caution in interpreting the observed associations as indicative of lower vulnerability among older adults.

### Limitations

Cross-sectional data do not allow us to disentangle age, period, and cohort effects. Instead, longitudinal analyses would be required to distinguish the relative contributions of early-life experiences of food and fuel rationing, ageing and disability processes, and contemporaneous shocks, such as the cost-of-living crisis and recent geopolitical turmoil, to patterns of food, fuel, and financial insecurity across adulthood. As none of the Scottish longitudinal datasets capture food and fuel insecurity, we relied on the JRF’s latest poverty dataset to compare and contrast multidimensional poverty between different age groups. Despite this shortcoming, the paper allows us to observe differences across adulthood which is especially relevant given recent attempts to cut fuel allowances for older people in the UK.

This analysis uses secondary data that was conducted exclusively online over a short period (11 days), which may have influenced the types of individuals who participated. While older adult internet use has steadily increased over time, older adults with lower income and lower education levels are less likely to access the internet and have digital literacy skills [43, 44]. Given the additional costs associated with internet access and suitable devices, the most vulnerable older adults will have been less likely to participate in this online survey [43]. As a result, the data does not fully capture the extent of food, fuel, and financial insecurity among the most deprived older populations in Scotland. This potential selection bias may lead to an underestimation of poverty measures, as those experiencing the most severe forms of deprivation are less likely to be included in the sample. This limitation is particularly salient given the already high prevalence of fuel insecurity observed in our findings. Future research should seek to capture the experiences of the most deprived older adults through more inclusive survey designs (e.g. in-person and telephone options) and, where appropriate, purposive sampling strategies targeting individuals likely to experience compounded disadvantage across the life course.

A combination of subjective and objective measures, while unavailable in this survey, would be ideal in surveying multidimensional poverty and health [45, 46]. Self-reported measures allow us to include people who are self-rationing food and fuel and likely help distinguish between those who may have limited financial resources but have robust family/community resources [20]. As highlighted in Supplementary Appendix 3, income-insecure older adults, by objective measures are less likely to self-report financial insecurity (averaging 35%) than younger adults (44%). This apparent discrepancy may partly reflect access to non-income-based resources, such as accumulated assets or informal family support, which can buffer financial strain without being captured in income measures. However, as unpacked above, there may be reporting barriers, such as normalization or internalized stigma that, especially among older adult populations, potentially result in underreporting or obfuscation of self-determined associations. Our measures of food and fuel insecurity are based on behavioural coping strategies, which may capture both temporary responses and more sustained hardship. Future research incorporating a combination of subjective and objective measures, ideally within longitudinal designs, could help address these limitations.

Due to small sample sizes, we could not include ethnicity in our models, nor were we able to create a model for older adults alone. Within some of the age disaggregation conducted, some confidence intervals remain higher than ideal due to sample sizes. More research is needed to explore the interaction of multidimensional poverty and ethnicity on health and wellbeing in Scotland. However, we ran the health model with and without controlling for pre-existing physical disability, mental health condition, and physical health and examined any changes in significance. Our findings were unique in that we explored the interaction of age, multidimensional poverty and health impacts. While exploring these impacts across adulthood, we avoided treating older adults as a single group and instead noted differences between those aged 65–74 and 75+.

An important next step is to extend this work within a capabilities framework or Alkire-Foster methodology, enabling a more explicit assessment of how material insecurities constrain individuals’ opportunities to achieve valued functionings in later life [19]. This would require richer data to capture capabilities directly and to inform context- and age-sensitive weighting of dimensions, thereby advancing a more theoretically grounded account of multidimensional disadvantage.

This study shows that food, fuel, and financial insecurity are widespread in Scotland during the cost-of-living crisis and are strongly associated with adverse physical, mental, and social health outcomes across adulthood. Although our study suggests that older age is generally associated with lower odds of negative health outcomes among materially secure groups, this association is substantially eroded by insecurity, with particularly high odds of health impacts observed among younger and middle-aged adults. Fuel insecurity emerged as highly prevalent and only partially aligned with financial insecurity, indicating that reliance on income-based measures alone would underestimate deprivation and its health consequences. Together, these findings underscore the importance of a multidimensional approach to poverty measurement and policy. They further highlight that current levels of insecurity reflect not only income constraints, but also structural barriers to accessing necessities, reinforcing the need for policy responses that directly address the material conditions under which households are attempting to meet their food and fuel needs. Addressing health inequalities in Scotland will require integrated social protection strategies that extend beyond income support, explicitly tackling food and fuel insecurity across the life course, with targeted interventions for both working-age and older adults.

## Supplementary Material

ckag089_Supplementary_Data

## Data Availability

This data are publicly available via the Joseph Rowntree Foundation website. Key points Multidimensional poverty is widespread and heterogeneous across adulthood in Scotland. Fuel insecurity is highly prevalent and insufficiently captured by income-based measures. Food and financial insecurity show strong age-income gradients, with younger and working-age adults most impacted. All insecurity dimensions are associated with adverse physical, mental, and social health impacts. A paradox emerges in later life whereby older insecure adults report fewer health impacts despite known health vulnerability.
