# Supplementary material for: Multidimensional poverty in Scotland and health across adulthood—the paradoxical associations with food, fuel, and financial insecurity in later life
Source: Eur J Public Health. 2026 Jun 19;36(4):ckag089. doi: 10.1093/eurpub/ckag089 (PMC13281941; doi:10.1093/eurpub/ckag089)
Supplement: ckag089_Supplementary_Data [file ckag089_supplementary_data.zip › ejph-2026-03-om-0217-File004.docx]

**Supplementary References**

41. Purdam K, Garratt EA, Esmail A. Hungry? Food Insecurity, Social Stigma and Embarrassment in the UK. Sociology. 2016 Dec 11;50(6):1072–88. doi:10.1177/0038038515594092

42. Slocombe H. Aged spaces in an era of austerity: Food bank use by older people. Area. 2023 Sep 24;55(3):407–15. doi:10.1111/area.12870

43. Remillard ML, Mazor KM, Cutrona SL, Gurwitz JH, Tjia J. Systematic Review of the Use of Online Questionnaires of Older Adults. J Am Geriatr Soc. 2014 Apr 17;62(4):696–705. doi:10.1111/jgs.12747

44. Hunsaker A, Hargittai E. A review of Internet use among older adults. New Media Soc. 2018 Oct 16;20(10):3937–54. doi:10.1177/1461444818787348

45. Tu G, Morrissey K, Sharpe RA, Taylor T. Combining self-reported and sensor data to explore the relationship between fuel poverty and health well-being in UK social housing. Wellbeing, Space and Society. 2022;3. doi:10.1016/j.wss.2021.100070

46. Manikas I, Ali BM, Sundarakani B. A systematic literature review of indicators measuring food security. Agric Food Secur. 2023 May 5;12(1):10. doi:10.1186/s40066-023-00415-7
