# Supplementary material for: Multidimensional poverty in Scotland and health across adulthood—the paradoxical associations with food, fuel, and financial insecurity in later life
Source: Eur J Public Health. 2026 Jun 19;36(4):ckag089. doi: 10.1093/eurpub/ckag089 (PMC13281941; doi:10.1093/eurpub/ckag089)
Supplement: ckag089_Supplementary_Data [file ckag089_supplementary_data.zip › ejph-2026-03-om-0217-File005.docx]

**Appendix 1 - Sociodemographic Characteristics of the JRF Poverty in Scotland 2023 Survey**

| Characteristic | Frequency (%^a^) |
| --- | --- |
| Age |  |
| 18–24 | 418 (10.7) |
| 25–34 | 664 (17.2) |
| 35–44 | 626 (15.3) |
| 45–54 | 740 (16.6) |
| 55–64 | 743 (16.8) |
| 65–74 | 652 (15.2) |
| 75+ | 360 (8.2) |
|  |  |
| Sex |  |
| Female | 2287 (48.1) |
| Male | 1900 (51.5) |
| Other | 16 (0.4) |
|  |  |
| Ethnicity |  |
| White | 3871 (95.4) |
| Ethnic Minority | 319 (4.3) |
|  |  |
| Income category |  |
| Low-income | 1275 (33.5) |
| Middle and/or high-income | 2455 (66.5) |
|  |  |
| Solo Dweller |  |
| Yes | 1083 (22.9) |
| No | 3120 (77.1) |
|  |  |
| Region |  |
| Eastern Scotland^b^ | 1316 (29.0) |
| South Western Scotland^c^ | 1212 (31.2) |
| North Eastern Scotland | 387 (8.9) |
| Highlands and Islands | 381 (8.9) |
| City of Edinburgh | 356 (10.0) |
| Glasgow City | 551 (12.0) |
|  |  |
| Physical Disability |  |
| Yes | 800 (18.4) |
| No | 3037 (74.5) |
| Someone else in the household | 302 (7.1) |
|  |  |
| Physical Health Condition |  |
| Yes | 1014 (23.1) |
| No | 2799 (69.1) |
| Someone else in the household | 329 (7.8) |
|  |  |
| Mental Health Condition |  |
| Yes | 1277 (31.0) |
| No | 2530 (61.1) |
| Someone else in the household | 319 (7.9) |

*^a^ Percentages are weighted*

*^b^ Not including Edinburgh*

*^c^ Not including Glasgow*
