# Supplementary material for: Multidimensional poverty in Scotland and health across adulthood—the paradoxical associations with food, fuel, and financial insecurity in later life
Source: Eur J Public Health. 2026 Jun 19;36(4):ckag089. doi: 10.1093/eurpub/ckag089 (PMC13281941; doi:10.1093/eurpub/ckag089)
Supplement: ckag089_Supplementary_Data [file ckag089_supplementary_data.zip › ejph-2026-03-om-0217-File006.docx]

**Appendix 2 - Regression with health impacts as the outcome and age and lower income in interaction, without controlling for the presence of physical disability, mental health condition, or physical health condition of the respondent or in the household**

|  | Negative Physical Health Impact | | Negative Mental Health Impact | | Negative Social Impact | |
| --- | --- | --- | --- | --- | --- | --- |
|  | Odds Ratio | (95% conf. interval) | Odds Ratio | (95% conf. interval) | Odds Ratio | (95% conf. interval |
| Fuel Security Status by Age |  |  |  |  |  |  |
| Secure 18-24 (ref) | 1.00 | | 1.00 | | 1.00 | |
| Secure 25–34 | 0.66* | 0.44–0.99 | 0.67 | 0.45–1.00 | 0.99 | 0.68–1.46 |
| Secure 35–44 | 0.66 | 0.43–1.01 | 0.56* | 0.37–0.84 | 0.92 | 0.62–1.38 |
| Secure 45–54 | 0.56* | 0.37–0.85 | 0.39* | 0.26–0.58 | 0.74 | 0.50–1.11 |
| Secure 55–64 | 0.31* | 0.20–0.49 | 0.17* | 0.11–0.27 | 0.43* | 0.28–0.65 |
| Secure 65–74 | 0.11* | 0.06–0.19 | 0.07* | 0.04–0.11 | 0.14* | 0.09–0.23 |
| Secure 75+ | 0.23* | 0.13–0.40 | 0.12* | 0.07–0.20 | 0.22* | 0.14–0.37 |
| **Insecure 18–24** | 1.17 | 0.77–1.78 | 2.71* | 1.66–4.44 | 2.46* | 1.59–3.78 |
| Insecure 25–34 | 1.50* | 1.03–2.18 | 1.88* | 1.25–2.84 | 2.74* | 1.86–4.03 |
| Insecure 35–44 | 1.27 | 0.88–1.83 | 1.41 | 0.95–2.08 | 1.85* | 1.28–2.67 |
| Insecure 45–54 | 1.40 | 0.98–1.99 | 1.15 | 0.79–1.67 | 2.22* | 1.55–3.17 |
| Insecure 55–64 | 1.04 | 0.73–1.49 | 0.66* | 0.46–0.95 | 1.37 | 0.97–1.96 |
| Insecure 65–74 | 0.40* | 0.27–0.59 | 0.25* | 0.17–0.37 | 0.61* | 0.42–0.89 |
| Insecure 75+ | 0.51* | 0.32–0.82 | 0.21* | 0.13–0.35 | 0.55* | 0.35–0.87 |
|  |  |  |  |  |  |  |
| Food Security Status by Age |  |  |  |  |  |  |
| Secure 18-24 (ref) | 1.00 | | 1.00 | | 1.00 | |
| Secure 25-34 | 1.05 | 0.72–1.52 | 0.63* | 0.44–0.90 | 1.08 | 0.76–1.52 |
| Secure 35-44 | 0.99 | 0.68–1.44 | 0.55* | 0.39–0.79 | 0.94 | 0.67–1.32 |
| Secure 45-54 | 0.99 | 0.69–1.41 | 0.41* | 0.29–0.57 | 0.89 | 0.64–1.24 |
| Secure 55-64 | 0.73 | 0.51–1.05 | 0.25* | 0.18–0.35 | 0.62* | 0.45–0.86 |
| Secure 65-74 | 0.25* | 0.16–0.38 | 0.09* | 0.07–0.14 | 0.25* | 0.18–0.35 |
| Secure 75+ | 0.42* | 0.27–0.66 | 0.10* | 0.06–0.15 | 0.25* | 0.17–0.38 |
| Insecure 18-24 | 4.06* | 2.57–6.41 | 2.20* | 1.29–3.76 | 2.82* | 1.77–4.50 |
| Insecure 25-34 | 3.47* | 2.33–5.16 | 1.96* | 1.27–3.02 | 3.08* | 2.06–4.62 |
| Insecure 35-44 | 4.37* | 2.86–6.69 | 2.16* | 1.35–3.45 | 3.02* | 1.96–4.64 |
| Insecure 45-54 | 5.72* | 3.75–8.74 | 2.92* | 1.79–4.76 | 5.70* | 3.58–9.07 |
| Insecure 55-64 | 5.37* | 3.35–8.59 | 1.44 | 0.88–2.35 | 3.36* | 2.09–5.40 |
| Insecure 65-74 | 2.78* | 1.61–4.81 | 0.68 | 0.39–1.17 | 1.78* | 1.03–3.07 |
| Insecure 75+ | 2.21* | 1.17–4.19 | 0.54 | 0.29–1.02 | 1.38 | 0.74–2.55 |
|  |  |  |  |  |  |  |
| Financial Security Status by Age |  |  |  |  |  |  |
| Secure 18-24 (ref) | 1.00 | | 1.00 | | 1.00 | |
| Secure 25-34 | 0.93 | 0.67–1.28 | 0.68* | 0.49–0.95 | 1.11 | 0.81–1.51 |
| Secure 35-44 | 0.89 | 0.64–1.24 | 0.58* | 0.42–0.81 | 0.87 | 0.63–1.18 |
| Secure 45-54 | 0.75 | 0.54–1.04 | 0.39* | 0.28–0.54 | 0.76 | 0.56–1.04 |
| Secure 55-64 | 0.59* | 0.43–0.82 | 0.24* | 0.18–0.33 | 0.57* | 0.42–0.78 |
| Secure 65-74 | 0.24* | 0.16–0.34 | 0.10* | 0.07–0.14 | 0.23* | 0.17–0.33 |
| Secure 75+ | 0.34* | 0.23–0.51 | 0.12* | 0.08–0.18 | 0.28* | 0.19–0.40 |
| Insecure 18-24 | 2.76* | 1.69–4.51 | 3.73* | 1.88–7.42 | 3.19* | 1.87–5.45 |
| Insecure 25-34 | 3.13* | 2.04–4.79 | 3.45* | 1.96–6.05 | 3.71* | 2.31–5.95 |
| Insecure 35-44 | 3.07* | 2.00–4.71 | 3.65* | 2.01–6.61 | 4.57* | 2.73–7.64 |
| Insecure 45-54 | 3.64* | 2.48–5.34 | 2.83* | 1.76–4.54 | 5.99* | 3.77–9.52 |
| Insecure 55-64 | 2.83* | 1.85–4.34 | 1.45 | 0.90–2.34 | 2.71* | 1.72–4.25 |
| Insecure 65-74 | 1.43 | 0.79–2.61 | 0.99 | 0.52–1.88 | 2.67* | 1.37–5.22 |
| Insecure 75+ | 10.30* | 2.89–36.70 | 0.77 | 0.28–2.08 | 1.91 | 0.68–5.38 |
|  |  |  |  |  |  |  |

** – Statistically significant, P < .05*

*Ref – reference group*

*Also controlled for sex, solo dweller status and location.*
