# Supplementary material for: Multidimensional poverty in Scotland and health across adulthood—the paradoxical associations with food, fuel, and financial insecurity in later life
Source: Eur J Public Health. 2026 Jun 19;36(4):ckag089. doi: 10.1093/eurpub/ckag089 (PMC13281941; doi:10.1093/eurpub/ckag089)
Supplement: ckag089_Supplementary_Data [file ckag089_supplementary_data.zip › ejph-2026-03-om-0217-File007.docx]

**Appendix 3 – Crosstabulation of income security and self-reported financial insecurity by age group**

|  | Self-Reported Financial Insecurity | |
| --- | --- | --- |
|  | No | Yes |
| Ages 18-24 |  |  |
| Income secure  Income insecure | 0.86 | 0.14 |
|  | 0.61 | 0.39 |
|  |  |  |
| Ages 25-34 |  |  |
| Income secure  Income insecure | 0.82 | 0.18 |
|  | 0.61 | 0.39 |
|  |  |  |
| Ages 35-44 |  |  |
| Income secure  Income insecure | 0.84 | 0.16 |
|  | 0.56 | 0.44 |
|  |  |  |
| Ages 45-54 |  |  |
| Income secure  Income insecure | 0.77 | 0.23 |
|  | 0.42 | 0.58 |
|  |  |  |
| Ages 55-64 |  |  |
| Income secure  Income insecure | 0.86 | 0.14 |
|  | 0.58 | 0.42 |
|  |  |  |
| Ages 65-74 |  |  |
| Income secure  Income insecure | 0.96 | 0.04 |
|  | 0.73 | 0.27 |
|  |  |  |
| Ages 75+ |  |  |
| Income secure  Income insecure | 0.98 | 0.02 |
|  | 0.79 | 0.21 |
